# Computational Aided-Molecular Imprinted Polymer Design for Solid Phase Extraction of Metaproterenol from Plasma and Determination by Voltammetry Using Modified Carbon Nanotube Electrode

**Published:** 2014

**Authors:** Farhad Ahmadi, Ehsan Karamian

**Affiliations:** a *Nano Drug Delivery Research Center, Faculty of Pharmacy, Kermanshah Universityof Medical Sciences, Kermanshah, Iran. *; b *Pharmaceutical Chemistry Department, Faculty of Pharmacy, Kermanshah University of Medical Science, Kermanshah, Iran.*

**Keywords:** Doping control, Metaproterenol, Computational molecular modeling, Molecularly imprinted polymer, Modified carbon nano tube electrode

## Abstract

A molecular imprinted polymer (MIP) was computationally designed and synthesized for the selective extraction of metaproterenol (MTP), from human plasma. In this regards semi empirical MP3 and mechanical quantum (DFT) calculations were used to find a suitable functional monomers. On the basis of computational and experimental results, acrylic acid (AA) and DMSO:MeOH (90:10 %V/V) were found to be the best choices of functional monomer and polymerization solvents, respectively. This polymer was then used as a selective sorbent to develop a molecularly imprinted solid-phase extraction (MISPE) procedure followed by differential pulse voltammetry by using modified carbon nanotube electrode. The analysis was performed in phosphate buffer, pH 7.0. Peak currents were measured at +0.67 V versus Ag/AgCl. The linear calibration range was 0.026–8.0 μg mL^-1^ with a limit of detection 0.01 μg mL^-1^. The relative standard deviation at 0.5 μg mL^-1^ was 4.76% (n*=5*). The mean recoveries of 5 μg mL^-1^ MTP from plasma was 92.2% (n=5). The data of MISPE-DPV were compared with the MISPE-HPLC-UV. Although, the MISPE-DPV was more sensitive but both techniques have similar accuracy and precision.

## Introduction

**Figure 1:**
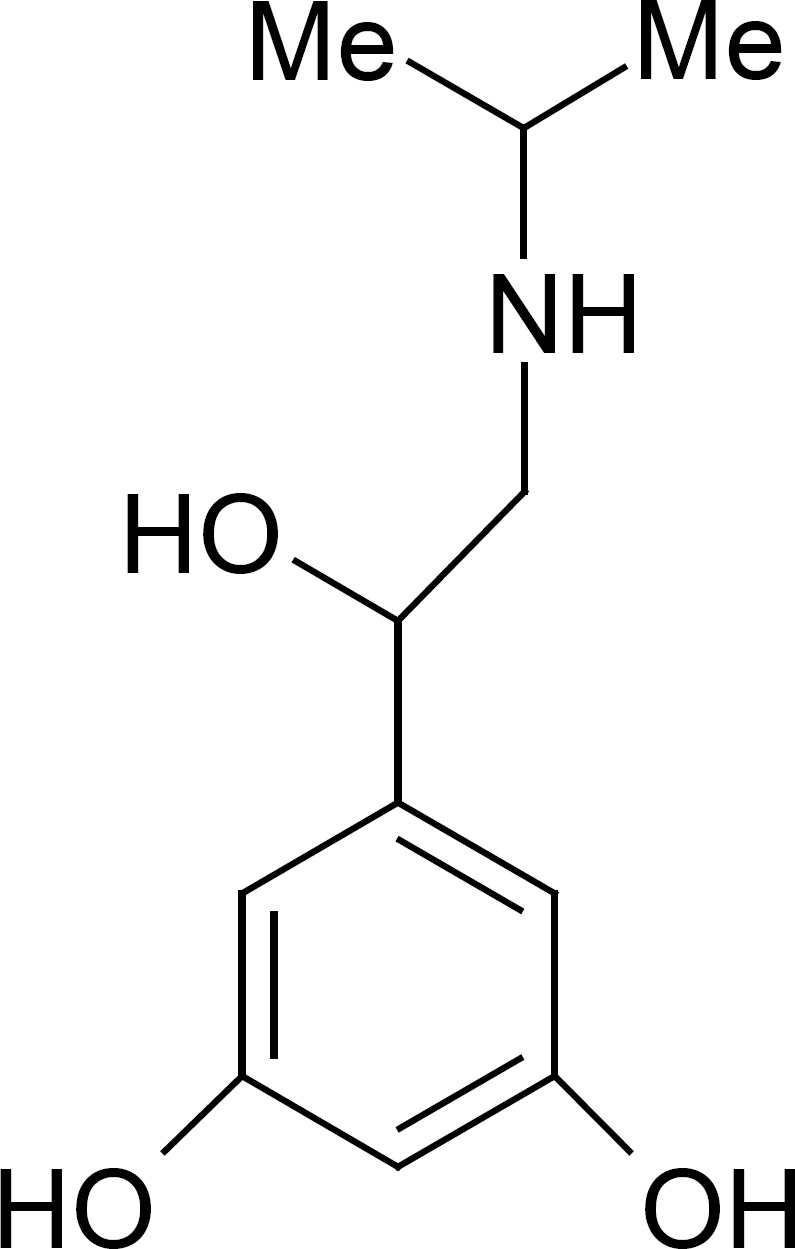
The structure of metaproterenol (MTP).

The World Anti-Doping Agency (WADA) has defined more than two hundred substances on the prohibited list that are forbidden either in- and out-of-competition or only in-competitions (1). These forbidden drugs are classified into nine categories; two groups of analytes prohibited in particular sports and three forbidden methods (*i.e*., enhancement of oxygen transfer, physical and chemical manipulation and gene doping). In this regards the β_2_-agonists are classified as doping agents in sports. β_2_-agonists belong to the phenyl β-ethanolamine compounds (2), which have different substituent groups on the aromatic ring as well as on the terminal amino group. Based on the substituent groups on the aromatic ring, β_2_-agonists can be divided into three groups: phenolic group including: salbutamol, ractopamine, bamethane, isoxsuprine and ritodrine, *etc*.; aniline group containing: clenbuterol, clenproperol, mabuterol, cimaterol, cimbuterol, brombuterol, *etc*.; and resorcinol group, such as terbutaline, metaproterenol and fenoterol. At Summer Games in Athens in 2004; the analysis and medical justification of β_2_-agonists was an important challenge of International Olympic Committee Medical Commission and this problem is may be remained as a challenge for future competitions (3). Therefore, the analysis of β_2_-agonists in biological fluids is very importance for doping control. Metaproterenol (3,5-dihydroxy-a-[(isopropylamino) methyllbenzyl alcohol, MTP, Figure 1) is a potent β-adreno receptor agonist with a rapid onset of action when given by inhalation therapy. To our knowledge, few expensive reports existed for determination of MTP in plasma samples such as HPLC-fluorescence detector (4), GC-Mass (5) and liquid chromatography/atmospheric pressure chemical ionization mass spectrometry and tandem mass spectrometry (6). Sane *et al*., reported a colorimetric method for determination of MTP in pharmaceutical preparation (7). As it is classified as doping agents and there is not an inexpensive, fast, and simple method for monitoring of MTP in plasma, therefore, develop of accurate analytical methods for selective trace determination of MTP is of interest. Generally, for determination of trace residue of drugs in plasma samples a pre-enrichment step is needed. Although, solid-phase extraction (SPE) is a common procedure for extraction of drugs from plasma samples (8), but due to the very low selectivity, the co-elution of many interference species on conventional sorbents is a serious problem. For overcoming this problem, molecularly imprinted polymers (MIPs) have been developed as a selective sorbents for solid phase extraction (MIP-SPE) of analytes from different matrixes. However, to obtain an efficient polymer it is needed to synthesize a wide range of polymers with trial and error, and during experiments, the hazardous compounds are so harmful for people’s health. Besides, in the synthesis of MIPs, most of the standard templates are expensive. Due to this limitation, in our laboratory we used the computational methods as an alternative approach for the rational design of MIPs (9-11). In this regards the density functional theory (DFT) method was widely used to select functional monomers or porogenic solvents among a set of traditional chemicals by calculating the energy difference (ΔE) (12-14). Recently, the combination of MIP-SPE with voltammetric methods was developed as an interest and effective system for fast, accurate and economic analysis of electroactive drugs in plasma samples. For example in our laboratory, we have designed and synthesized a very selective MIP for SPE of Acetazolamide from plasma before determination by differential pulse voltammetry (10). Also, Gholivand and Khodadian have designed and synthesized a MIP for extraction of Methocarbamol from plasma. After MISPE, the drug was determined by both differential pulse voltammetry (DPV), and high performance liquid chromatography (HPLC) with UV detection (15). They show that MISPE-DPV is more sensitive but both techniques have similar accuracy and precision. The library studies show that techniques which are used mostly for determination of β_2_-agonists in biofluids are: capillary zone electrophoresis (16), liquid chromatography with mass spectrometry (MS) (17), gas chromatography with MS (18), and HPTLC (19). Electrochemical methods are the preferred methods for the detection of various drugs (20) such as β_2_-agonists, because most of the β_2_-agonists can be oxidized at bare or modified electrodes. In recent years, the application of multiwall-carbon nanotubes (MWCNTs) in electrode modification has received remarkable attention in electrochemistry (21-26). The modification of electrode substrates with MWCNTs would result in low detection limits, reduced over potentials and resistance to surface fouling and therefore MWCNTs have been claimed as electrocatalysts (20, 22, 23, 26). In this article, the MP3 semi empirical and DFT-quantum mechanical calculations were used for design of a MTP-imprinted polymer. The MIP was then synthesized to develop a MISPE procedure for the selective extraction of MTP from human plasma. The MTP was determined by differential pulse voltammetry on a glassy carbon electrode modified with MWCNTs. The electro behavior of MTP, optimum MISPE and measurement conditions were described by DPV using glassy carbon and glassy carbon modified with MWCNTs electrodes.

## Experimental

*Materials*

Metaproterenol hemisulfate salt, salbutamol, methocarbamol and clenbuterol were purchased from Sigma-Aldrich (Louis, USA). A standard solution of 100 µg mL^-1 ^of metaproterenol was prepared by dissolving an appropriate amount of the drug in methanol. This solution was stored at dark and 4 °C. Other diluted solutions were prepared by dilution from the standard solution. Acrylic acid (AA), ethylene glycol dimethacrylate (EGDMA), 2,2'-azobis(isobutyronitrile) (AIBN) and HPLC grade solvents such as: dimethylformamid (DMF), dimethylsolfoxid (DMSO), methanol (MeOH) and acetonitrile (AN) were purchased from Merck (Darmstadt, Germany). The multiwall carbon nanotube was purchased from Sigma-Alderich (Louis, USA). All monomers were distilled under the reduced pressure to remove their stabilizers before use. Human plasma samples were obtained from healthy volunteers and stored at -20 °C until use. All other chemicals, were of analytical reagent grades and used without further purification.

*Instrumentation*

All voltammograms were recorded using a Metrohm Computrace instrument model VA 797. Measurements were carried out on a GC or GC-MWNT modified electrodes in a three-electrode arrangement. The auxiliary electrode was a wire of platinum; and an Ag/AgCl electrode was used as reference electrode. All measurements were carried out at room temperature. A Metrohm-827 pH-meter (Switzerland) was used for pH adjustments. High performance liquid chromatography (HPLC) was performed on a KNAUER liquid chromatograph instrument. A Windaus two-channel peristaltic pump model D-38678 was used to pump solvents during MISPE experiments. The polymerization was performed in a thermo bath model FINPCR (ALB64) at 55 ˚C temperature.

*Fabrication of GC/MWCNT electrode*

The bare glass carbon electrode (GCE) was polished by using 0.3 μm alumina slurry on micro-cloth pads and rinsed carefully with double distilled water. Then the GCE was activated by polarization at +1.7 V for 90 s and at −1.7 V for 90 s like to work of Zhu *et al.*, (27). In this step for preparation of MWCNT-GCE, 10.0 μL of suspended MWCNT in DMF (0.5 mg mL^-1^) was inserted onto the surface of pretreated GCE. The electrode was allowed to dry at room temperature, and then electrochemically activated by CV between +1.7 and −1.7 V at scan rate of 50 mV s^-1^. 

*The DPV measurements*

The differential pulse voltammetric measurements for determination of MTP were carried out in 10 mL phosphate buffer solution (0.1 M, pH=7.0) at room temperature (25 °C). The differential pulse voltammograms were recorded from 0.2 to 1.2 V with scan rate of 50 mV s^-1^, pulse amplitude of 50 mV, and pulse time of 40 ms at GC and/or GC/MWCNTs electrodes. 

*Computational approach*

All calculations have been carried out by using Gaussian 03 (28) software package. The computational method developed, was based on the MP3 semi empirical method and density functional theory (DFT) to locate the most stable template-monomer complexes. The interaction energies and ∆*E*, were calculated through the equation (1): 

equation (1) $$\mathrm{\Delta E}=E_{\left(\mathrm{MTP}-\mathrm{monomer}\right)}-(E_{\mathrm{MTP}}+{\mathrm{\Sigma E}}_{\left(\mathrm{monomer}\right)})$$

Beacause polymerization is occurred in solution, we must take the effect of solvent, or solvation into account, in energy calculations because it leads to changes in energy and stability of the template-monomer complexes. Methods for evaluating the solvent effect may broadly be divided into two types: those describing the individual solvent molecules and those that treat the solvent as a continuous medium (29-31). Continuum models (32), which are more popular, consider the solvent as a uniform polarizable medium with a dielectric constant of *ε*, while the solute is placed in a suitably shaped cavity in the medium (33). In this section, the polarizable continuum model, developed by Tomasi and co workers (34-36), was used to study the effect of solvent in energy calculations. In this study, electronic energies were first calculated using MP3 method. The most stable structures from this step were further optimized through the DFT method at B3LYP/6-31G(d,p) level. In order to introduce the effect of solvent in energy calculations, the polarizable continuum model was used.

*Preparation of MIP and NIP*

MIP was prepared by using bulk polymerization method by dissolving 0.4 mmol of MTP in 2.0 mL of DMSO:MeOH (90:10 %V/V) solvents (HPLC grade) in a 15.0 mL glass tube equipped with a rubber cap. Then, 1.6 mmol of functional monomer AA, 8.0 mmol of cross-linker EGDMA, and 0.1 mmol of AIBN as initiator were added. The ratio of MTP vs functional monomer used was 1/4. The solution was deoxygenated and saturated with dry nitrogen for 5 min and the tube was sealed under nitrogen (99.99%). Finally, the test tube was placed in a thermo bath at 55 °C for 12 h. The hard polymer was grounded in laboratory under wet conditions. The resultant polymeric particles were collected and the template molecule was extracted from the polymer with 95:5 (%V/V) MeOH: acetic acid mixture in a Soxhlet extraction system during 24 h. The reference non-imprinted polymer (NIP) was prepared by using the same procedure in the absence of template molecule. 

*MISPE-DPV for plasma sample*

According to our previous work, 50 mg of each dry polymer (MIP and NIP) was packed into empty 5 mL SPE-cartridges between two polyethylene frits (37). Prior to each extraction, cartridges were conditioned with 1.0 mL of MeOH and 2.0 mL of phosphate buffer (1.0×10^-3 ^mol L^-1^, pH 4.0). To exactly 0.9 mL of human plasma that spiked with a known concentration of MTP, 0.2 mL of AN was added and centrifuged at 5000r pm (4 ^◦^C) for 10 min to precipitate proteins. The clear supernatant was then diluted to 2.0 mL with phosphate buffer (pH 4.0) and then percolated through the MIP or NIP cartridge. The cartridge was washed with 1.0 mL of 99.5:0.5 (%V/V) phosphate buffer:MeOH and dried by passing of pure N_2_ gas. Then, the drug was eluted from the cartridge by using 1.0 mL of MeOH. The elution fractions was then collected and diluted to 10 by 0.1 M phosphate buffer (pH=7) and transferred to the voltammetric cell. The voltammogram was recorded as described in general procedure section.

*Clean-up procedure using C18-SPE cartridges*

In order to compare MIP with classical reversed-phase SPE sorbents, a 3 mL-volume C18-SPE cartridge (Restek U.S., Bellefonte, PA) was used to extract the drug from a plasma sample. The procedure was performed according to our previous work with some modification (10). The SPE conditions were optimized and general procedure for plasma sample was as follows: the cartridge was preconditioned with 2.0 mL of MeOH, followed by 2.0 mL of binary mixture of H_2_O:AN (90:10 %V/V). To exactly 0.9 mL of human plasma spiked with a known concentration of MTP, 0.1 mL of AN was added and centrifuged at 5000 rpm (4 ^◦^C) for 15 min to precipitate proteins. The protein-free supernatant was passed through the conditioned cartridge by a flow rate of 1.0 mL min^−1^. The cartridge was washed with 0.5 mL of H_2_O:AN (90:10 %V/V) and the drug was eluted with 1.0 of MeOH. Elution fraction was collected in a test tube and analyzed by proposed procedure.

*MISPE-HPLC-UV*

The method of MISPE-HPLC-UV was roughly the same of MISPE-DPV procedure with some differences in last step: the dried sample was dissolved in 200 μL of mobile phase of HPLC, and 20 μL of this solution was analyzed by HPLC at the wavelength of 275 nm for metaproterenol determination. A reversed phase C18 column (Eurospher 100-5C18 column (250×4.0 mm *i.d*.)) and a 20 μL injection loop were used. The mobile phase was 50:50 (%V/V) mixture of 0.02 mol L^-1^ phosphate buffer (pH 3.0):AN with a flow rate of 1.0 mL min^-1^. 

## Results and Discussion

*Voltammetric study*

**Figure 2:**
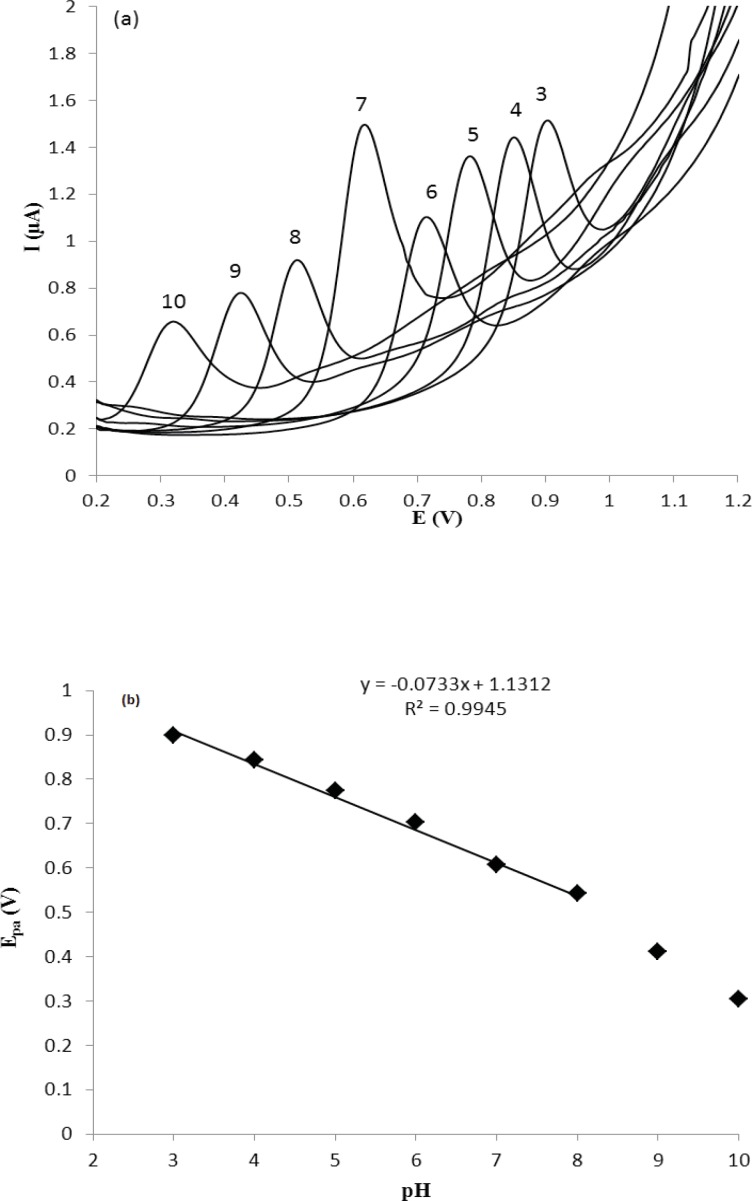
(a) Effect of pH on the DP voltammetric current of 5.0 µg mL^-1^ MTP at GC electrode; (b) The dependences of anodic peak potential E_pa _versus pH for MTP at GC electrode. The conditions were as follows: 10 mL of electrolyte containing phosphate buffer-MeOH (90:10 %V/V), scan rate 50 mV s^-1^.

**Figure 3:**
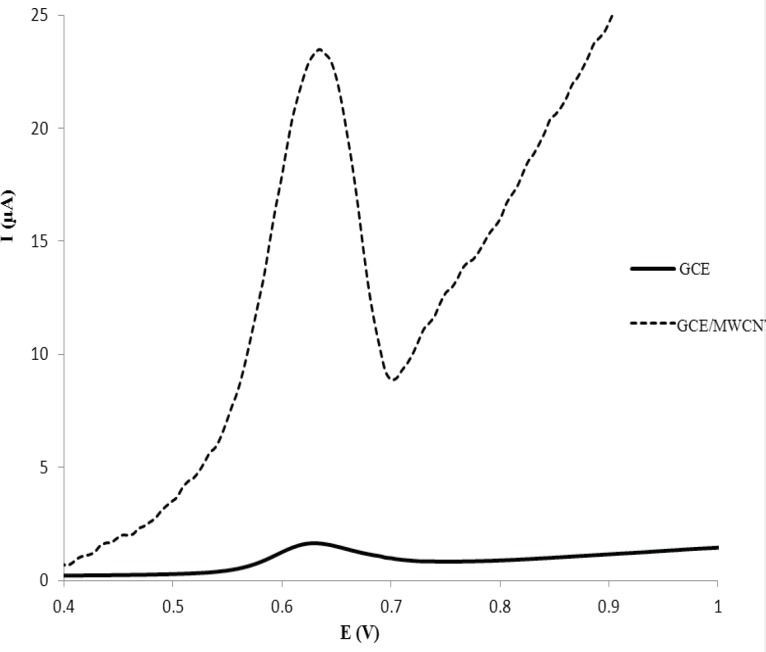
Differential pulse voltammograms of 6.0 µg mL^-1^ MTP in 0.1 M phosphate buffer (pH 7) on: GCE (black line); and GC/MWNT (green line) electrodes

The cyclic voltammetric behavior of several β_2_-agonists on bare carbon paste, nafion modified carbon paste and graphite nanosheet modified glassy carbon electrodes in BR buffer, pH 2-12, were studied by two researcher groups (38, 39). Shen and coworkers reported that the MTP can be oxidized irreversibility at graphite nanosheet modified electrode with enhanced peak current in compared with naked glassy carbon electrode (39). In this section, we studied the differential pulse voltammetric (DPV) behavior of MTP at both glassy carbon (GC) and modified nano carbon glassy carbon (GC/MWCNT) electrodes in the pH range of 3.0-10. Figure 2a shows the DPV response of the drug on a bare GC electrode in B.R buffer in the pH range of 3.0-10.0 within the potential window of 0.2–1.2 V. From Figure 2a, it can be seen that the oxidation peak current of MTP increases with increasing the pH value until it reaches 7.0, and then the oxidation peak current decreases when the pH increases further. It has been reported that the pKa value of MTP is 6.8 (40). When pH is between 3 and 7, the hydroxyl groups in MTP well not ionized, which will decrease the oxidation capacity of MTP (41). When pH increases from 7.0 to 10.0, the increased hydroxyl ion in the solution might also decrease the adsorption capacity of the MTP (41). Therefore, pH 7.0 was chosen as the optimal experimental condition. The relationship between the peak potential and pH was also investigated and the results were shown in Figure 2b. For MTP a linear shift of E_pa_ towards negative potentials with the regression equation of E_pa_(V) = -0.0733pH + 1.131 (R=0.994) for the oxidation, was observed in the pH range of 3.0 - 8.0. The slope of the equation is roughly close to the theory value of 60 mV pH^−1^ for two electrons and two protons process, indicating that the electrochemical redox of MTP at GC electrode should be a two electrons and two protons process (41). The same DPV results for MTP at GC/MWCNT under the same experimental conditions were observed, except in Faradic oxidation current of MTP. In other words, the Faradic response of MTP at the GC/MWCNT electrode is much higher than which observed at the bare GC electrode (Figure 3). The current enhancement for MTP on modified GC electrode might be attributed to the large surface area of MWCNT (42). The influence of electrochemical parameters known to affect the differential pulse voltammograms viz., pulse amplitude, pulse time and scan rate were studied. The variables of interest were studied over the ranges 10–200 mV for pulse amplitude, 100–2000 ms for pulse wide and 10–250 mV s^−1^ for scan rate. It was found that the peak height increased by increasing the scan rate, however, the peak current decreased as the pulse time increased. To acquire voltammograms of relatively high sensitivity and well shaped waves with relatively a narrow peak width, values of 50 mV, 40 ms and 50 mV s^−1^ were chosen for pulse amplitude, pulse time and scan rate, respectively.

*Theoretical selection of functional monomer and polymerization solvent*

Widely attempts have been carried out for using of semi-empirical and *ab initio *computational methods to the design of high selective molecularly imprinted polymers (43-46). Generally the semi-empirical methods such as AM1 or MP3 describe the properties of the polymer system based on an approximation of the electron distribution in the molecules studied, and gave a clearer picture of the interactions that are the basis of the MIP technology.

**Figure 4:**
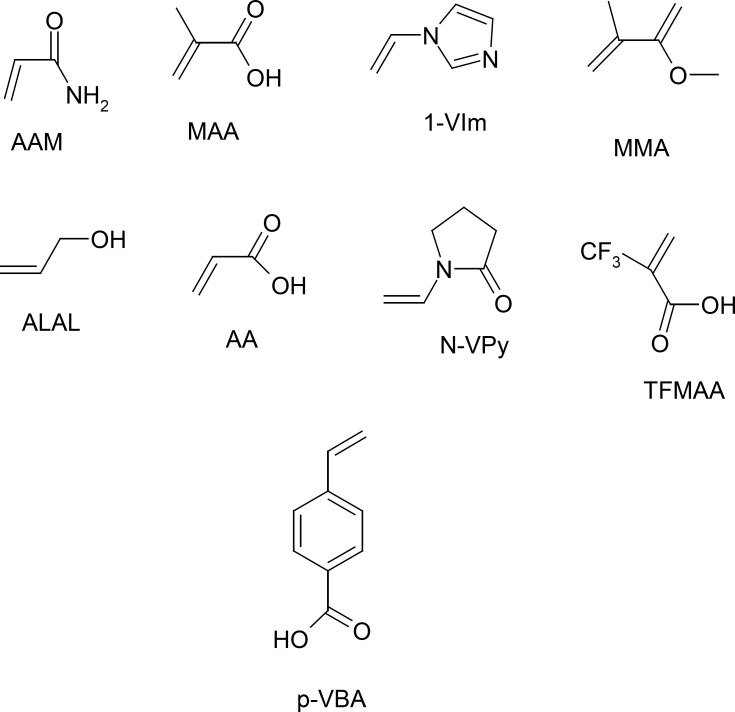
The virtual library of functional monomers: acrylamide (AAM), methacrylic acid (MAA), 1-vinylimidazole (1-VIm), methyl methacrylate (MMA), alylalcohol (ALAL), acrylic acid (AA), N-vinyl pyridine (N-VPy), trifluoromethacrylic acid (TFMAA), and p-vinyl benzoic acid (p-VBA).

While, the *ab initio calculations *can be describe a system with higher accuracy at minimal computational cost. In other words the mechanical quantum calculations yield considerably better representations of the non-covalent interactions present in the system under study than when using less rigorous simulations. Therefore, in this section we applied the semi empirical MP3 and mechanical quantum based on DFT, at the B3LYP level with 6-31-G(d,p) basis set for conformation optimization 9 of the most popular monomers such as: N-vinyl pyridine (N-VPy), acryl amid (AAM), acrylic acid (AA), alylalcohol (ALAL), methacrylic acid (MAA), three fluoromethacrylic acid (TFMAA), para-vinyl benzoic acid (p-VBA), 1-vinylimidazol (1-VIm), and methyl methacrylate (MMA), (Figure 4). The conformation of template, functional monomers and template-monomer complexes were first optimized to the lowest energies using the fast MP3-semiempirical method. For each selected functional monomer, the most stable template–monomer complexes (1:1), interaction energies, ΔE, were calculated through Equation (1). According to the quantum mechanical calculations the monomer with highest binding energy to the template is mostly suitable for the preparation of MIP. As it is observed from Table 1 the order of 1:1 complex energy are as follows: MTP-AAM> MTP-TFMAA> MTP –N-VPy> MTP -MAA> MTP –p-VBA> MTP -MMA> MTP-ALAL> MTP-1-VIm> MTP-AA. 

**Table 1:** Interaction energies of 1:1, 1:2, 1:3 and 1:4 MTP– monomer complexes in gas phase which calculated by semi empirical MP3 method

**Compound name**	**MTP-(monomer)**_1_ **(Kj mol** ^-1^ **)**	**MTP-(monomer)**_2_ **(Kj mol** ^-1^ **)**	**MTP-(monomer)**_3_ **(Kj mol** ^-1^ **)**	**MTP-(monomer)**_4_ **(Kj mol** ^-1^ **)**
1-VIM	-9.228	-25.689	-55.797	-75.651
AA	-22.199	-46.288	-68.488	-78.472
AAM	-9.228	-17.962	-28.876	-26.840
ALAL	-11.499	-42.459	-69.084	-75.855
MAA	-14.994	-28.294	-45.489	-61.161
MMA	-18.939	-38.957	-58.270	-72.959
N-VPy	-20.844	-33.657	-58.755	-57.315
P-VBA	-16.797	-27.719	-38.671	-70.039
TFMAA	-6.121	-36.352	-57.524	-43.623

**Figure 5:**
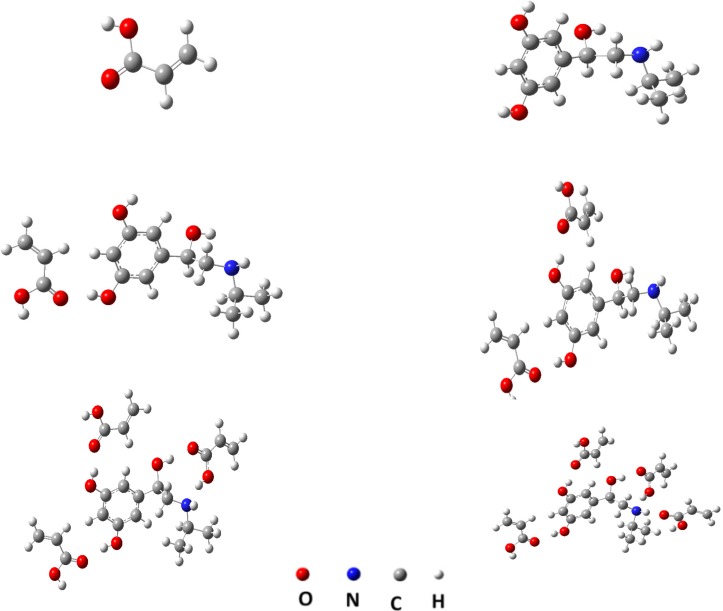
Computationally derived structures of AA, MTP and 1:1, 1:2, 1:3 and 1:4 complexes of MTP with AA using DFT method at B3LYP/6-31G(d,p) level

Hence, AA is more likely to possess a greater rebinding and higher selectivity than MIP prepared with other monomers. The same method was adopted to calculate the binding energy between MTP and monomers, with the molar ratios of 1:2, 1:3 and 1:4. The results showed that, the highest stability was obtained for the 1:4 mole ratio of template–monomer complexes. The metaproterenol-AA complex was further optimized by using a more accurate quantum mechanical calculation. Figure 5 shows, for example, the optimized geometries of 1:1, 1:2, 1:3 and 1:4 of MTP-AA complexes predicted by DFT method at B3LYP/6-31G(d,p) level. The interaction energies were also calculated in different solvents by using the PCM model. From the data listed in Table 2, it is concluded that toluene, DMSO and MeOH are the most favorable solvents for MIP preparation, but due to the very low solubility of metaproterenol hemisulfate salt in toluene, the DMSO was considered as progon for preparation of MIP. However, the metaproterenol hemisulfate salt was not freely soluble in DMSO, therefore, the 90:10 %V/V of binary mixture of DMSO: MeOH was used as suitable binary solvents for preparation of MIP.

**Table 2:** The interaction energy of 1:1, 1:2, 1:3 and 1:4 MTP-(AA)_n_ in several solvents which calculated by DFT and B3LYP/6-31G (d, p) method.

**solvent**	**ΔE**_1:1_ (Kj mol^-1^)	**ΔE**_1:2_ (Kj mol^-1^)	**ΔE**_1:3_ (Kj mol^-1^)	**ΔE**_1:4_ (Kj mol^-1^)
Water	-2.343	-13.211	-42.872	-69.032
Nitromethane	-35.406	-54.799	-85.650	-99.046
DMSO	-39.355	-59.604	-88.568	-110.159
Methanol	-13.656	-58.987	-88.357	-107.495
Toluene	-37.993	-85.855	-119.965	-152.696
THF	-15.239	-38.313	-66.368	-98.614
Acetonitrile	-23.641	-41.709	-66.188	-93.673

*Optimization of MISPE procedure*

*Effect of pH*

**Figure 6:**
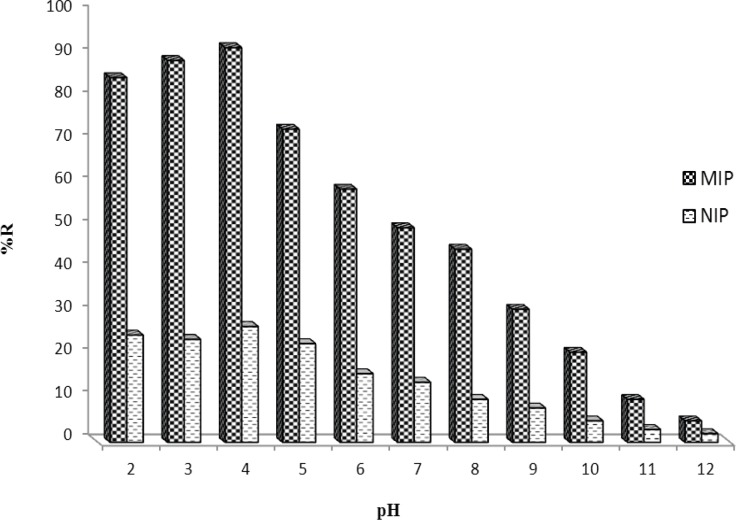
Effect of pH on the MISPE of 3.0 µg mL^-1^ MTP and follows by DPV-GC/MWCNT.

To have a better interaction between MIP and target molecule in aqueous media, the effect of sample pH should be studied. The effect of buffer pH on MTP binding was investigated over the pH range 2.0-12.0 (Figure 6). Clearly, the binding behavior of MTP was not greatly affected at pHs < 5.0. However, at more alkaline pHs the recovery of the drug was decreased as pH increased which may be due to the deprotonation of carboxylic acid of the monomer (pKa≈4.3) or hydrolysis of the drug. The pH 4.0 was selected for subsequent MISPE experiments.

*Selection of washing solvent*

**Figure 7:**
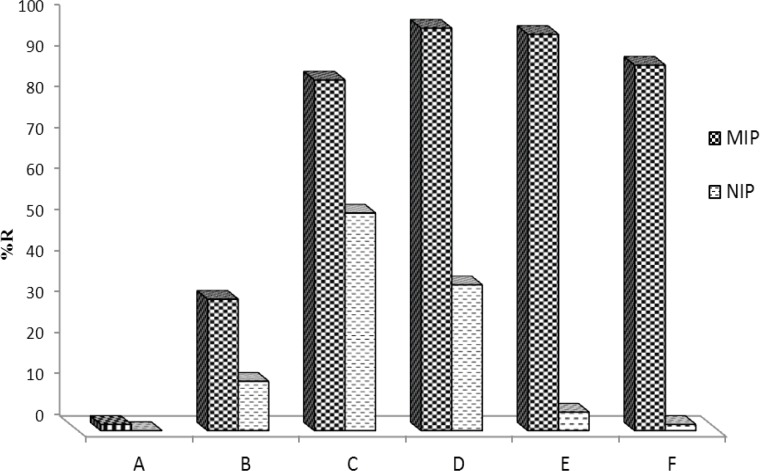
Effect of different washing solvent/solutions on MIP and NIP's recovery. (A) MeOH, (B) Water, (C) Acetonitrile, (D) phosphate buffer solution (pH=4.0), (E) phosphate buffer solution (pH=4.0)+0.5% MeOH and (F) phosphate buffer solution (pH=4.0)+1.0% MeOH.

Washing MIP is a crucial step in developing a MISPE procedure because the general procedure for reducing problems of nonspecific adsorption is the selection of a proper washing solvent prior to elution (47). Thus, in this section several washing solvents/solutions were tested to develop a proper washing step (Figure 7). Solvents such as water, methanol, acetonitrile, phosphate buffer and their binary mixtures were tested. As can be seen, the best washing solution was phosphate buffer pH=4 containing a small amount of methanol. With increasing percentage of methanol, however, analyte recovery decreased. However, addition of up to 0.5% methanol to phosphate buffer could be permitted without considerable loss of recovery. The final MIP extraction method uses an aqueous wash with 1.0 mL of phosphate buffer (pH=4):MeOH (99.5:0.5 %V/V) with the loss of analyte being less than 2.5%. This composition of phosphate buffer and methanol also serves the maximum difference between MIP and NIP's recovery. 

*Selection of elution solvent*

**Figure 8:**
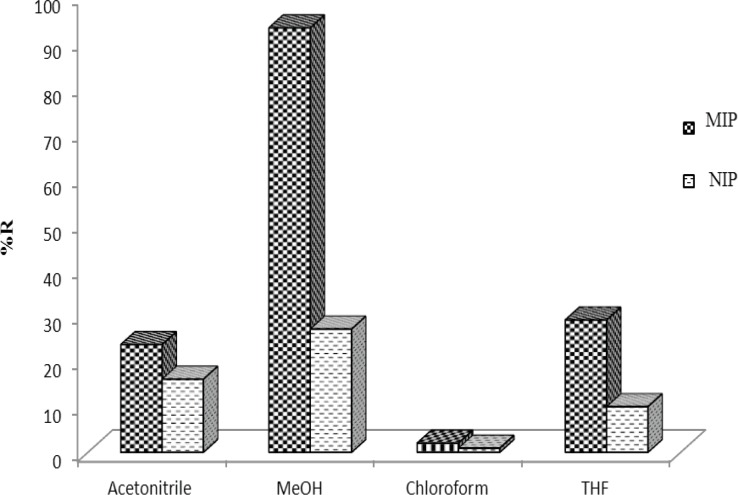
Effect of elution solvent on MIP and NIP's recovery

The strong imprint–analyte interaction must be destroyed to reach a high extraction recovery; therefore a series of experiments was performed to study the elution solvent composition. Solvents such as methanol, acetonitrile and chloroform were tested as eluents. Among these solvents the best recoveries were obtained using by MeOH (Figure 8). Therefore, 1.0 mL of MeOH was used as elution solution throughout the present study.

*The selectivity test*

**Figure 9:**
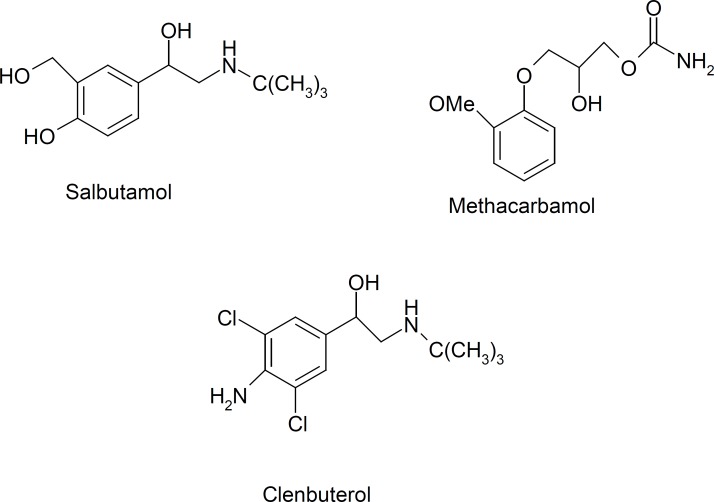
The structures of salbutamol, clenbuterol and methocarbamol

To evaluate the selectivity of the synthesized MIP, salbutamol, clenbuterol and methocarbamol were considered in this section (Figure 9). In several batch experiments, the distribution ratio (*K*_d_) and selectivity coefficients (*α*) were calculated and are listed in Table 3.

The *K*_d_ values were calculated by using the equation:

equation (2) $$K_{d}=\frac{(C_{i}-C_{f})V}{C_{f}m}$$

Where *V*, *C*_i_, *C*_f_ and *m* represent the volume of the solution (mL), drug concentration before and after adsorption (µg mL^-1^) and mass of the polymer, respectively. The selectivity coefficient (α) is defined as:

equation (3) $$\alpha =\frac{K_{d(\mathrm{MTP})}}{K_{d(\mathrm{Foreign Compounds})}}$$

The results from Table 3 clearly suggest that the unique shape of the template molecule plays an important role in its selective binding to the MIP. 

**Table 3:** Distribution ratio (*K*_d_) and selectivity coefficient (*α*) values for imprinted and Non-imprinted polymers

**Compound**	**MIP**		**NIP**	
	***K***_d_	***α***	***K***_d_	***α***
Metaproterenol	298.7		57.4	
Salbutamol	112.3	2.65	58.9	0.97
Methocarbamol	79.6	3.75	67.7	0.84
Clenbuterol	194.2	1.54	61.2	0.94

*Validation of the method*

The MISPE-DPV-GC/MWCNT method for the determination of MTP was validated by determining its performance characteristics regarding to linearity, repeatability, and precision. To test the DPV response linearity, a series of plasma standard solutions of MTP in the concentration range 0.026–8.0 µg mL^-1^ were analyzed as mentioned in section 2.7 (at least ten samples that covering the whole range were used). The relationship between peak current (I_p_, µA) and concentration (C_MTP_, µg mL^-1^) was linear for MTP according to the equation I_p_ = 3.872C_MTP_ + 0.021; (R^2^ =0.996). The LOQ and LOD are the concentrations at which the signal to noise ratio is 3 and 10, respectively. The limit of detection (LOD) and the limit of quantification (LOQ) were 0.01 and 0.026 µg mL^-1^, respectively. The RSD value for intraday assay reproducibility at 0.5 µg mL^-1^ solution (n = 5) was found to be 4.76% indicating good repeatability of the method. The mean recoveries of 5 μg mL^-1^ MTP from plasma was 92.2% (n=5)

*Interference study*

Under the selected conditions for determining the drug, the interference of some species commonly present in biological media was examined by analyzing a standard solution of 0.5 μg mL^-1 ^MTP. The tolerable limit of foreign species was considered with the relative error less than 3.5% (48). The results are listed in Table 4. These results clearly show that MIP can be used as a good adsorbent for cleaning-up of complex samples to improve the selectivity of the DPV method.

**Table 4:** Tolerable concentration ratios with respect to metaproterenol for some interfering substances using DPV and MISPE-DPV

**Interfering substances**	**DPV**	**MISPE-DPV**
Starch	450	2500
Urea	250	1900
Uric acid	200	1000
Glucose, sucrose, ascorbic acid	100	750
Creatinine, glycine	80	700
Histidine	7	300
Glutathione	1	250

*Evaluation of MISPE-DPV for plasma samples*

**Figure 10:**
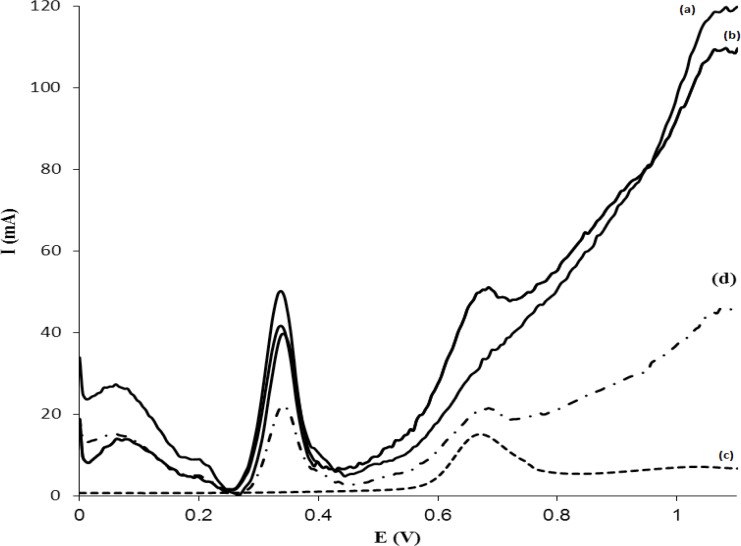
Voltammograms of (a) a blank of plasma, (b) a plasma spiked with 6.0 µg mL^-1^ MTP without any sample treatment and MISPE, (c) a plasma spiked with 3.0 µg mL^-1^ MTP after plasma pretreatment, MISPE, washing off with 1.0 mL of phosphate buffer–MeOH (99.5:0.5, %V/V, 0. 1 M, pH = 4) solution, and (d) a plasma spiked with 3.0 µg mL^-1^ MTP after plasma pretreatment, SPE using C-18 as sorbent

The applicability of MISPE-DPV method was tested for human plasma samples. The results are shown in Figure 10. The voltammogram of Figure 10a represent the blank plasma, while the voltammogram Figure 10b represents the plasma that spiked with 6.0 µg mL^-1^MTP without any sample pretreatment and MISPE. Figure 10c shows the voltammogram of a plasma sample containing 3.0 µg mL^-1^ of MTP that is pretreatment and passed through the MISPE cartridge and analyzed by DPV-GC/MWCNT. As it is observed, when a sample was passed through the MISPE with a washing step (using 1.0 mL Buffer: MeOH), all compounds were washed off from MIP cartridge and MTP was selectively retained on MIP. In addition to compare the performance of the proposed MISPE procedure with commercial SPE sorbents, a voltammogram was recorded after a clean-up procedure using C18-SPE cartridge and is shown in Figure 10d. The recovery of the drug was about 49% with a higher number of contaminating peaks compared to that recorded for MIP cartridge, which demonstrates the usefulness of the MISPE procedure. From these results it is concluded that, MIP exhibits a high selectivity for MTP. As a control experiment, extraction on NIP was investigated in similar conditions. Results showed that NIP extraction led to a low recovery (less than 25%). This shows that, most of the templates were cleaned up with matrix components during the washing step. Also, it is worth noting that a cartridge packed with 50 mg of MIP can be used to clean-up at least 4 plasma samples with a very scanty noticeable deterioration in performance. After each extraction, the MISPE cartridge was regenerated by washing with 3.0 mL 90:10 (%V/V) mixture of MeOH:acetic acid, 1.0 mL of MeOH at a flow rate of 1.0 mL min^−1^. Also, in order to validate the methodology and confirm its potential in routine monitoring of MTP, the same samples were subjected to HPLC analysis. Table 5 compares the results of the analysis of MTP between the two methods. As can be seen, the methods show similar accuracy and precision at the concentrations tested as revealed by the t-test and f-test, respectively. The proposed procedure was used for the determination of MTP in one suspicious sample. The sample for extraction of MTP was subjected to MISPE procedure by using 1.0 mL of washing solvent and the extract was analyzed by DPV by using modified GC/MWCNT electrode. MTP was not detectable in the suspicious sample.

## Conclusion

The results of the present study show that the computer assisted-design of MIP can be used as a powerful tool to screen functional monomers for a specified template molecule. The calculations predicted that AA was the best choice of functional monomer for the preparation of MIP. The polymer was then synthesized and used as a selective adsorbent to develop a molecularly imprinted solid-phase extraction (MISPE) procedure for selective extraction of metaproterenol from human plasma before differential pulse voltammetry (DPV). The combination of MISPE and differential pulse voltammetry by using GC/MWCNT electrode considerably enhanced the selectivity of the voltammetric technique. The developed MISPE-DPV-GC/MWCNT method exhibited good analytical performance in terms of selectivity, sensitivity, reproducibility and accuracy for quantification of MTP in complex biological samples such as human plasma. 
